# A pavlovian model of the amygdala and its influence within the medial temporal lobe

**DOI:** 10.3389/fnsys.2015.00041

**Published:** 2015-03-18

**Authors:** Maxime Carrere, Frédéric Alexandre

**Affiliations:** ^1^LaBRI, UMR 5800, CNRS, Bordeaux INP, Université de BordeauxTalence, France; ^2^Inria Bordeaux Sud-OuestTalence, France; ^3^Institut des Maladies Neurodégénératives, UMR 5293, CNRS, Université de BordeauxBordeaux, France

**Keywords:** amygdala, pavlovian conditioning, hippocampus, perirhinal cortex, infralimbic cortex, acetylcholine

## Abstract

Recent advances in neuroscience give us a better view of the inner structure of the amygdala, of its relations with other regions in the Medial Temporal Lobe (MTL) and of the prominent role of neuromodulation. They have particularly shed light on two kinds of neurons in the basal nucleus of the amygdala, the so-called fear neurons and extinction neurons. Fear neurons mediate context-dependent fear by receiving contextual information from the hippocampus, whereas extinction neurons are linked with the medial prefrontal cortex (mPFC) and involved in fear extinction. The computational model of the amygdala that we describe in this paper is primarily a model of pavlovian conditioning, but its architecture also emphasizes the central role of the amygdala in the MTL memory processes through three main information flows. (i) Thalamic and higher order sensory cortical inputs including from the perirhinal cortex are received in the lateral amygdalar nucleus, where CS-US associations can be acquired. (ii) These associations are subsequently modulated, in the basal nucleus of the amygdala, by contextual inputs coming from the hippocampus and the mPFC. Basal fear and extinction neurons indicate the currently valid association to their main targets including in the MTL and the mPFC. (iii) The competition for the choice of the pavlovian response is ultimately performed by projection of these amygdalar neurons in the central nucleus of the amygdala where, beyond motor responding, a hormonal response, including cholinergic modulation, is also triggered via the basal forebrain. In turn, acetylcholine modulates activation in the basal nucleus and facilitates learning in the hippocampus. Based on biologically founded arguments, our model replicates a number of biological experiments, proposes some predictions about the role of amygdalar regions and describes pavlovian conditioning as a distributed systemic learning, binding memory processes in the MTL.

## 1. Introduction

Since the seminal studies by Pavlov, respondent conditioning has been extensively studied, both at the behavioral and neurophysiological levels. Particularly, fear conditioning, contextual fear extinction and renewal are prototypical protocols that brought much knowledge about the corresponding behaviors and neuronal circuitry. In these protocols, an unconditioned stimulus (US) corresponds to a biologically significant stimulus (e.g., an electric shock) that automatically triggers a fear response (e.g., freezing). In fear conditioning, an initially neutral stimulus (the CS: conditioned stimulus) is repeatedly paired with the US during an acquisition phase. Subsequently, the CS presented alone triggers a fear response, which can be interpreted as an anticipation of the occurrence of the US by the CS. For example, Herry et al. (2008) describes this protocol in mice, with the first conditioned response appearing as soon as the third CS-US association. Conditioning is not necessarily related to a salient stimulus but can also involve the context in which conditioning occurs. In contextual fear conditioning, the spatial context (the chamber in which the rat is placed before receiving an electric shock) can become a strong predictor of the US (Fanselow, 2000).

Fear extinction occurs when, in some context, the CS is presented but not followed by the US. In this case, CS might lose its ability to trigger a fear response but this is a long process (9–12 trials needed in Herry et al. (2008) to extinguish the association) and also rather fragile: when the animal is put back in the original conditioning environment, the CS-US association is renewed immediately. This indicates that extinction is a context-dependent process and that, during extinction, the association is not forgotten but only inhibited (Maren, 2005). Renewal only consists in releasing the inhibition.

The neuronal circuitry of pavlovian conditioning has benefited from extensive studies, from the molecular to the behavioral levels. The amygdala plays a central role in that circuitry, implementing pavlovian conditioning as a learning process to extract emotional values of stimuli in the world and to trigger corresponding emotional responses. It is composed of several nuclei with distinct roles and physiological properties. Following most of the literature, three nuclei will be more particularly considered here: the lateral nucleus (LA), the basal nucleus (BA) and the central nucleus of the amygdala (CeA). In summary, the former two nuclei share some properties with the cortex (composed of excitatory glutamatergic principal neurons) and integrate sensory information, while the CeA nucleus is more similar to the striatal regions of the basal ganglia (inhibitory gabaergic neurons) (LeDoux, 2007) and is responsible for the motor, hormonal and autonomic expression of pavlovian responses.

Pavlovian processes are supposed to result from the interactions between the amygdala and other cerebral structures, particularly in the Medial Temporal Lobe (MTL). Accumulated experimental evidences about these interactions contribute to a better understanding of the inner amygdalar processes and of their role within the MTL.

Concerning sensory input, it is widely acknowledged that LA is responsible for learning CS-US association in fear conditioning (LeDoux, 2007). LA receives sensory input from the thalamus and the cortex (including pain-related information). Thalamic input brings crude sensory information (like a pure tone); cortical processing builds more elaborated sensory information (that might correspond to more structured CS) along its ventral axis including key regions of the MTL, like the perirhinal cortex. Fear responses in LA are rather short (100 ms reported in Burgos-Robles et al., 2009). As shown in Pendyam et al. (2013), longer CS will elicit (via LA) a sustained activity in the pre-limbic region (PL) of the medial prefrontal cortex (mPFC) that projects in turn to BA where more complex associations can be learned.

BA is the region of the amygdala that has benefited from the largest expansion in the recent evolutionary process (Cardinal et al., 2002). It integrates information from LA and is a key structure at the interface with other cerebral regions. BA plays a major role in contextual fear conditioning (Goosens and Maren, 2001; Biedenkapp and Rudy, 2009), particularly due to its afferences from the hippocampus, essential for learning to integrate cues in their spatial and temporal context (Eichenbaum et al., 2012; Carretero-Guillén et al., 2013). BA is also widely reported to be involved in extinction, particularly due to its afferences from the infralimbic (IL) division of the mPFC (Sierra-Mercado et al., 2011), a region reported to keep track of the recent reward history (Wallis, 2007). These roles were particularly well-corroborated by the observation in Herry et al. (2008) of two distinct populations of neurons in BA, called fear and extinction neurons, differentially connected to the hippocampus and the IL and respectively involved in contextual fear conditioning and extinction.

In addition to its central role in providing emotional significance of events to structures responsible for instrumental conditioning (Cardinal et al., 2002; Balleine and Killcross, 2006) (particularly the ventral striatum and the mPFC), BA also participates in a fundamental learning process in the MTL, favoring the transfer of cortical information from the perirhinal cortex to the entorhinal cortex and the hippocampus (Paz and Paré, 2013), to create in this latter structure an episodic memory of the current situation, based on its emotional significance.

CeA is primarily considered as the motor pole of the amygdala, triggering pavlovian motor responses. In the case of fear conditioning, Ciocchi et al. (2010) reports projections from LA and BA nuclei, sometimes passing through masses of inhibitory intercalated cells (ITC), reaching in the lateral subdivision of CeA, so-called CeLOn and CeLOff neurons. These neurons respectively participate in the excitation and inhibition of the medial subdivision of CeA (CeM), driving fear response. Direct projections from ITC to CeM are also reported to participate in the inhibition of fear response (Amano et al., 2010; Lee et al., 2013). Freezing behavior is triggered via the periaqueductal gray (PAG) and this motor response is generally considered in experimental works as a marker of fear expression and its measure is used to evaluate the level of conditioning (LeDoux, 2007; Herry et al., 2008; Ciocchi et al., 2010; Sierra-Mercado et al., 2011). Another component of pavlovian responses involves neuromodulators and neuropeptides (Lee et al., 2013), introducing global factors (for example related to the level of attention, stress, novelty, etc. of the corresponding episode) in the elaboration of pavlovian conditioning. Among multiple roles attributed to neuromodulators (including some still in discussion), the release of acetylcholine (ACh) by the basal forebrain has been proposed in Yu and Dayan (2005) to be a marker of the level of uncertainty of the environment. Concerning its impact, ACh is characterized by a role in memory process, by switching the hippocampus in the storage mode (Hasselmo, 2006) and by its ability to modify the balance of activation between BA and LA: particularly a high level of ACh was experimentally associated in fear conditioning to a higher activation in the BA region (Calandreau et al., 2006).

At the moment, the diversity and complementarity of the roles of amygdalar nuclei and regions have been poorly incorporated in computational models, whereas they seem critical in light of the recent physiological studies. Early models of pavlovian conditioning were mainly interested in defining an associative learning rule, to relate sensory inputs to a faithful prediction of US. Some are based on the Rescorla-Wagner competitive error-driven learning rule (Rescorla and Wagner, 1972), that modifies the associational weights as a function of the error of prediction of the US. Others draw inspiration from the Makintosh rule (Mackintosh, 1975) and lay emphasis on the associative history of the sensory cues. As each kind of rules best explains specific aspects of pavlovian conditioning, hybrid models have also been proposed (Le Pelley, 2004), integrating in a single learning rule specific processings on the CS and US information. All these models can be considered behavioral, in the sense that they mainly insist on the accuracy of US prediction and not on a realistic representation of information in the inner neuronal mechanisms. For example in many models (Schmajuk and DiCarlo, 1992; Kruschke, 2001), the sensory input is a unique vector of neurons, with some undifferentiated neurons representing “the context.”

With the advance of neurobiological knowledge about pavlovian conditioning, some models were developed to describe the interplay between the amygdala, the mPFC, the posterior cortex and the hippocampus (Armony et al., 1997; Meeter et al., 2005; Pauli et al., 2011; Moustafa et al., 2013) but do not differentiate the BA and LA regions. Others integrate more biophysical details (Li et al., 2009; Kim et al., 2013; Pendyam et al., 2013), but they remain at the level of simple CS-US association in LA. Recently, Vlachos et al. (2011) proposed a model integrating experimental observation in BA by Herry et al. (2008) of fear and extinction neurons but the model restricts amygdalar contribution to the BA nucleus.

None of these existing models are differentiating LA and BA nuclei and particularly their specific contributions to different MTL pathways. We have designed an integrated model of the amygdala where each main nucleus has been modeled such that it is possible to understand and differentiate its interplay with other regions of the MTL (the hippocampus and the perirhinal cortex) and also with specific regions of the mPFC (IL). This model, replicating a number of recent experimental results in electrophysiology, underlines the complementary roles of the BA and LA nuclei in the exchanges with these regions and the critical role of the ACh neuromodulation.

## 2. Materials and methods

### 2.1. Network architecture and functioning rules

Our modeling study uses the DANA library for neuronal representation and computation (Rougier and Fix, 2012) and is coded in Python.

**Figure 1:**
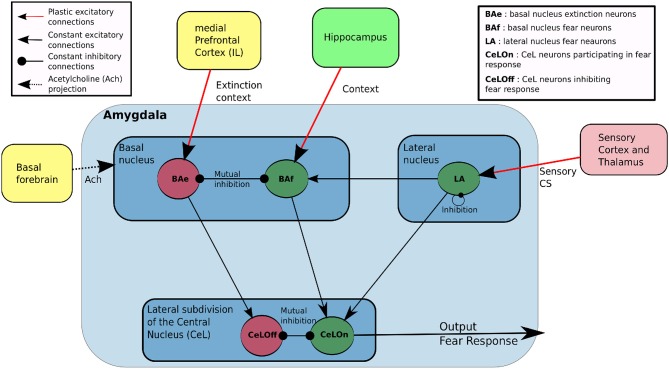
**Main features of our model of the amygdala**. LA and BAf are two fear neurons populations, which differ by their afferent connections. LA learn to predict fear based on sensory input from cortex and thalamus, and projects to BAf and CeLON. BAf receive contextual inputs from Hippocampus and sensory-based prediction from LA, resulting in a prediction based on both sensory or contextual information. BAe is a population of extinction neurons receiving contextual inputs from medial Prefrontal Cortex (Infralimbic cortex, IL) during extinction. BAe and BAf are in mutual inhibition and respectively project to CeLOn and CeLOff populations in CeA. CeLOn and CeLOff are also in mutual inhibition. CeLOn activity is considered as the level of US prediction of the model.

Though our study takes special care to model neuronal dynamics in the amygdala, it also includes a basic representation of the cortex (this term in the model refers to the spectrum of sensory inputs from the thalamus to the perirhinal cortex, as reported above), the hippocampus, the mPFC (including IL) and the basal forebrain (including cholinergic neurons), as depicted in Figure 1. Importantly, this figure also indicates information flows implemented between these structures. At the implementation level, one connection between two neuronal structures consists of a full connectivity between neurons of the input and output populations.

Special efforts were made to keep a reduced number of parameters and to test that the model was not too sensitive to their change (cf. Table 1 for a summary of all the parameters mentioned throughout the paper).

**Table 1:** **Parameters describing network architecture and parameters used in activation and learning rules**.

**Parameter**	**Meaning**	**Value**
**ARCHITECTURAL PARAMETERS**		
input_size	Size of input vectors from cortex, hippo, IL	10
la_size	Number of neurons in la	10
baf_size	Number of neurons in baf	10
bae_size	Number of neurons in bae	10
CeLOn_size	Number of neurons in CeLOn	1
CeLOff_size	Number of neurons in CeLOff	1
**EQUATION PARAMETERS**		
τ	Amygdala neurons time constant	0.05
α	Modulates learning speed in LA, BAf and BAe	1
il_tau	Time constant for ACh equation	5
noise_level	% of neurons intrinsic noise	1
θ	Neurons input threshold	0.3
ACh_min	Minimum of ACh level	1
ACh_max	Maximum of ACh level	2.5
ACh_strength	Modulates effect of ACh concentration on BA	0.5
ACh_uncertainty_strength	Modulates effect of uncertainty on ACh concentration	5
wmin	Minimum for modifiable weights initialization	0.01
wmax	Maximum for modifiable weights initialization	0.05
Cel_input_W	Constant weights from BAf, BAe and LA to Cel	0.2
LA_to_BAf_W	Constant weights from LA to BAf	0.1
LA_inhib_W	Constant weights inside LA	0.1
Cel_inhib_W	Constant weights between CelOn and CelOff	0.25
BA_inhib_W	Constant weights between BAf and BAe	0.05

The amygdala is represented by five different neuronal populations. In addition to a population of neurons representing the lateral nucleus (LA), the basal nucleus is represented by a population of fear neurons (BAf) and a population of extinction neurons (BAe) in mutual inhibition, as described in Herry et al. (2008). The central nucleus (CeA) also possesses two populations, to model CeLOn and CeLOff neurons triggering and inhibiting fear responses as reported in Ciocchi et al. (2010).

The cortex and hippocampus provide sensory inputs, respectively to LA and BAf. They are represented by input vectors (*Cortex* and *Hippo*), fed by an input flow depending on the learning protocol, as described in Section 2.4. Similarly, IL provides a contextual input *IL* to BAe, as a vector of the same size as *Hippo*, intended to represent contexts of extinction from the monitoring of reward history (Wallis, 2007).

Other variables are made available throughout the amygdala. *US* is a boolean variable indicating if a fearful US has been received. *ERR* is the error of prediction reported in Li and McNally (2014) to be computed in the PAG and sent back to the amygdala. *ACh* is the tonic level of ACh broadcast by the basal forebrain to all the network (McGaugh, 2004).

The output of the system is elaborated by the mutual inhibition between CeLOn and CeLOff neurons and the resulting activity in the CeLOn neurons, reported to be responsible for triggering the fear response via the medial subdivision of CeA (Ciocchi et al., 2010), will be considered here as the level of prediction of the US. All these relations between neuronal structures are consistent with bibliographic information reported in the section above and are depicted in Figure 1.

For each neuron *i* in an amygdalar population *Amyg* receiving an input from a cerebral structure or another amygdalar population (both kinds referred to as *Input*), its level of activation is evaluated through two main variables, *V* representing the membrane potential and *U* the firing rate, as classically defined with the mean-field formalism:

$$\begin{array}{l} \frac{dV_{i}^{Amyg}}{dt}=\left(-V_{i}^{Amyg}+F\left(\Sigma_{j}W_{ij}^{Amyg\;-\;Input}*U_{j}^{Input}\right)\right)/\tau \\ \;U_{i}^{Amyg}=noise\left(sigmoid\left(V_{i}^{Amyg}\right)\right)-\Sigma_{k}W_{ik}^{Amyg\;-\;Inhib}*U_{k}^{Inhib} \end{array}$$

τ is a time constant defining the dynamics of activity evolution and set to 0.05 throughout all the experiments. *F* is a non-linear threshold function:

F(U)=max(min_value, U−θ)

where *min_value* is a small value, set to 10^−3^ and θ is set to 0.3. A sigmoid (non-linear) function is applied to *V* to obtain the firing rate *U* and a *noise*() function is added to represent intrinsic neuronal noise, using a uniform distribution centered on the value *sigmoid*(*V*). Results reported here are robustly obtained with an interval length of 1% of *sigmoid*(*V*). Yet raising noise up to 20% does not alter network performance on the different paradigms. Dynamics are still similar with even higher noise, but network predictions become less precise.

*U*^*Input*^_*j*_ is the firing rate of the *j*th neuron of the *Input* population, rectified to positive values and *W*^*Amyg*−*Input*^_*ij*_ is the value of the synaptic weight between this neuron and the neuron *i* considered in the equation. The weights are modified according to the learning rules defined in Section 2.2 below.

Principal neurons in BA and LA are glutamatergic neurons and all the *W*^*Input*^ weights are considered excitatory. Inhibitory weights *W*^*Inhib*^ coming from inhibitory populations *Inhib* and influencing the evaluation of *U* in amygdalar populations are intended to integrate several cases. GABAergic CeLOn and CeLOff neurons are reported to be directly in mutual inhibition (Ciocchi et al., 2010). Mutual inhibition is also mediated by local inhibitory neurons between BAf fear neurons and BAe extinction neurons (Herry et al., 2008), and between LA glutamatergic neurons (Lee et al., 2013). All these cases of mutual inhibition might also be viewed as a global effect at the population level and will not be considered for learning below.

### 2.2. Learning rules

Plasticity is implemented on connections from Cortex to LA, and from Hippocampus and IL to BAf and BAe, respectively. The other weights are kept constant. The initial values of all the weights are drawn from an uniform distribution with an interval length of 0.04, centered on 0.03 for learning weights and centered on 0.2 for constant excitatory weights from LA or BAf to CeLOn and from BAe to CeLOff, and on 0.1 from LA to BAf. Inhibitory weights are centered on 0.25 within LA and between CeLOn and CeLOff and on 0.05 between BAf and BAe.

Concerning LA and BAf, both engaged in learning to predict fear (respectively from a feature encoded in the cortex or a context encoded in the hippocampus), learning obeys a Rescorla-Wagner like rule:

$$\frac{dW_{ij}^{Post\;-\;Pre}}{dt}=ERR*US*\alpha *U_{j}^{Pre}*U_{i}^{Post}$$

where *Pre* and *Post* are the presynaptic and post-synaptic populations, α is a constant learning coefficient (set to 1.0 for LA, BAf and BAe post-synaptic neurons) and *US* is the value of the US, received as defined in the learning protocol described in Section 2.4 below. *ERR* = (US − *U*_*CeLOn*_) is the prediction error and implements the main idea of the Rescorla-Wagner rule: weights stabilize when the output of the network *U*_*CeLOn*_ is able to predict the actual US, else they are modified as a function of the prediction error. The present equation, designed for fear neurons, also implies that these neurons will only learn when the fearful US is received (if not, *US* = 0 and *dW* = 0).

The learning rule of connections between IL and extinction neurons in BAe is designed to track changes in the accuracy of prediction of the US and is sensitive to negative values of the prediction error *ERR*, when the US is predicted but not coming (as the main intention is to report extinction).

$$\frac{dW_{ij}^{BAe\;-\;IL}}{dt}=-ERR*\alpha *U_{i}^{BAe}*U_{j}^{IL}$$

In the experiments reported below, *U*_*IL*_ is designed to be active only when the recent history of US arrival indicates an extinction context, as it has been proposed that IL is computing such statistics (Wallis, 2007). This results in preventing IL-BAe connections from learning an extinction context in the case of stochastic US occurrence (Yu and Dayan, 2005) and from unlearning when *ERR* is positive.

### 2.3. The role of neuromodulation

Based on information flows described above, LA, BAf, and BAe populations predict a fearful US or an extinction context and activate CeLOn or CelOff populations with a degree of confidence depending on the degree of matching between their actual input and their learnt experience. In this simple view, the resulting prediction is only a function of the indirect competition between LA and BA populations, accumulated in CeA. In our model, we propose, in accordance to biological observations (Calandreau et al., 2006), that cholinergic modulation is going to play an additional role in this competition, primarily by modulating the levels of activation in BA. Accordingly, *U*_*BAf*_ and *U*_*BAe*_ are going to be computed as described above and multiplied by a value representing the tonic level of ACh, *ACh*.

$$\begin{array}{l} \;ACh=ach\_strength\;*\;(baseline+ach\_uncertainty\_strength\\ \;*\;noise\left(sigmoid\left(V^{ACh}\right)\right))\\ dV^{ACh}/dt=\left(-V^{ACh}+F\left(\left|ERR\right|\right)\right)/\tau_{ACh} \end{array}$$

where *baseline* is the baseline level of tonic ACh (set to 1.0), τ_*ACh*_ is the constant of time set to 5, *F* is the threshold function and *ERR* the prediction error defined above. *ach_strength* modulates the effect of ACh concentration on BA activities, and is set to 0.5. *ach_uncertainty_strength* modulates the effect of the recent uncertainty *V*^*ACh*^ on ACh concentration, and is set to 5. This simple formula is intended to represent the level of known uncertainty (or stochasticity) of the environment, as this role is attributed to ACh in Yu and Dayan (2005). In short, these authors argue that in case of high stochasticity (as can be measured by frequent errors of prediction, whatever their sign), the decision should not rely on precise cues but rather promote the role of the context, hence the interest for ACh to favor BA receiving more contextual information as compared to LA receiving well-learnt sensory cues from the cortex.

### 2.4. Defining learning protocols

**Figure 2:**
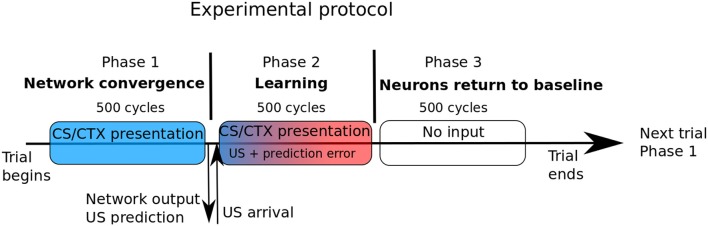
**In our experimental protocol, each trial is composed of three phases of identical duration**. In the first phase, only the sensory CS and/or the context is shown to the network. The second phase is the learning phase, and begins with US arrival. Prediction error is computed based of the network output at the end of phase 1. During phase 3, no input is presented and amygdalar neurons return to their baseline level of activation.

The model has to be activated to represent behavioral episodes including sensory events, motor responses and neuronal activation taking place in space and time. The goal of the experiments reported in the next section is to reproduce protocols described in experimental papers and to compare both model responses and internal activations to that reported in the papers, including in the case of some manipulations on the sensory inputs as well as on internal factors. We explain here how this procedure is implemented, concerning model activation and monitoring. Since this study is dedicated to pavlovian learning, the critical aspect is about representation in space and time of the sensory inputs. Concerning time, each behavioral episode is divided in three phases (see Figure 2). In the first phase, the external input is set on the *Cortex*, *Hippo* and *IL* vectors and remains for 500 cycles, for network activity stabilization. At the end of this phase, the activity in the CeLOn neuron gives the prediction of US by the network.

In the second phase, the US is given (possibly with value 0 in case of noUS) and remains for 500 cycles. At the very beginning of this phase, the error of prediction *ERR* is computed and the learning weights and level of ACh are updated. In the third phase with 500 cycles, no input are given and the network comes back to a rest state, ready for the next episode. Except for the simple case of extinction (US is anticipated but not occurring), we do not experiment variability of events in time. In our model, cortical, hippocampal, and IL inputs are represented by a vector of units representing sensory features and contexts. In these structures, in normal conditions, an input will correspond to a vector with one strongly activated unit (level of activation set to 1 in *Hippo* and *IL* and to 1.5 in *Cortex*) and the other units set to randomized values uniformly drawn between 0 and *n* = 1/*size_of_the_vector*. The strong activation is intended to represent salient features captured by an attentional process (as it is often the case in pavlovian conditioning) and the other fluctuating smaller values represent noise or non-significant details. It is hypothetized that learning could also perform well with lower levels of activation but would require more trials.

## 3. Results

In all the experiments reported in this paper, the architecture of the network was defined as follows: Input vectors *Cortex*, *Hippo*, and *IL* are of size 10 and LA, BAf, and BAe populations are composed of 10 neurons. For these simple experiments on fear conditioning, there are only one CeLOn and one CeLOff neuron.

The goal of the experiments is to run our model on specific protocols related to pavlovian learning and to compare its activation with observations reported in experimental papers, specifically emphasizing in a first set of experiments the complementary roles of the BA and LA nuclei in the exchanges with other MTL regions and in the second set, the critical role of the ACh neuromodulation.

### 3.1. Extinction-renewal experiment

The activation of LA, BAf, and BAe populations has been contrasted in the simulation of the classical pavlovian paradigms of fear conditioning, fear extinction and renewal and compared to results reported in Herry et al. (2008) and Ciocchi et al. (2010).

In the acquisition phase, a sensory CS (input from the cortex to LA) and a context CTX1 (input from hippocampus to BAf) are presented together to the network (phase 1) and maintained until the arrival of the US (phase 2). This trial is repeated 11 times, to ensure both full fear acquisition and ACh return to baseline after acquisition. In the extinction phase, CS is paired with another context, CTX2 (phase 1), without any US presentation in phase 2. This trial is repeated 14 times. For renewal, CS in paired again with CTX1 and the US prediction is observed at the end of phase 1.

**Figure 3:**
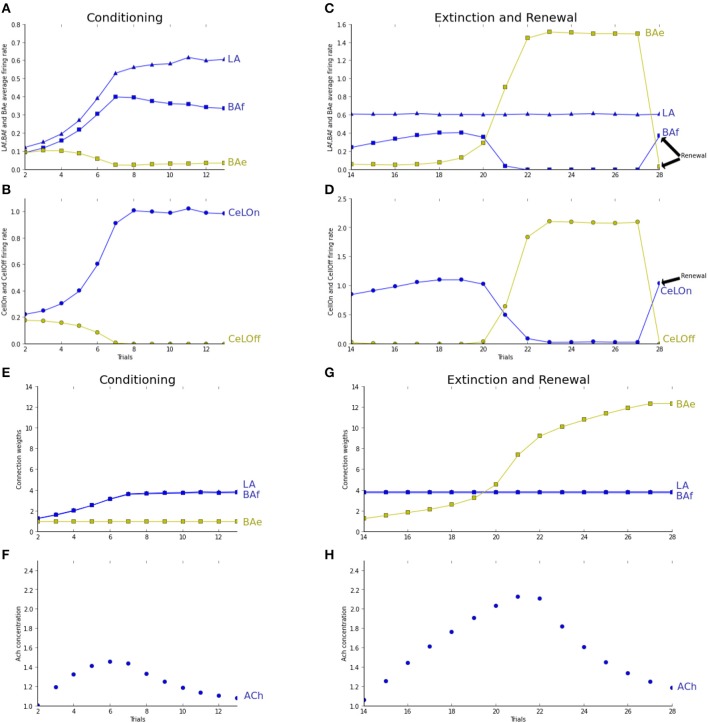
**Trial 1 (not shown here) was a trial without any stimulus presentation for network stabilization to baseline values**. Fear conditioning takes place from trial 2 to trial 10. Fear extinction from trial 11 to trial 21. Renewal is tested in trial 22. Graphs **(A–D)** are showing average activities at the moment of US arrival. Graphs **(E,F)** show average weights evolutions, normalized by their initial values. Graphs **(G,H)** show the evolution of ACh concentration over trials. **(A)** Both LA (blue triangles) and BAf (blue squares) activities increase during conditioning. BAe activity (green squares) decreases due to BAf inhibition. After a few trials, BAf reaches a maximum, then decreases weakly, due to ACh decrease. **(B)** Due to LA and BAf increase, CeLOn (blue circles) activity increases, and correctly predicts US arrival after conditioning. CeLOff activity (green circles) is inhibited by CeLOn **(C)** during the first trials, uncertainty rises, so does ACh, and BAf increases weakly. After a few trials, BAe activity starts to overcome BAf inhibition, and BAe strongly increases, which provokes BAf inhibition. LA firing rates remain steady. In the renewal context, IL does not provide any longer extinction information to BAe, which stops BAe firing and releases its inhibition on BAf. **(D)** During extinction, CeLOff activity increases with BAe increase and overcome CeLOn inhibition, still powered by LA firing. In the renewal trial, CeLOff is no longer sustained by BAe, and CeLOn activity is restored to conditioning value. **(E)** LA (blue triangles) and BAf (blue squares) weights are increasing during conditioning. BAe (green squares) remain steady. **(F)** Only BAe weights are increasing, with a higher rate as BAe starts winning the competition against BAf. **(G,H)** ACh increases with uncertainty, when the network makes prediction errors, and starts decreasing when the network correctly performs the task.

Figure 3 shows the responses of the model to the conditioning, extinction and renewal paradigms. In the conditioning phase, both LA and BAf populations are strengthening their connections with respectively the Cortex and Hippocampus, so that in a few trials, CeLOn activity is correctly predicting US arrival (Figure 3B). BAe and CeLOff activities are decreasing due to respectively BAf and CeLOn inhibition. Figure 3A shows that learning is similarly distributed between LA and BAf. Since uncertainty is high in the first trials, ACh concentration rises, allowing BAf to learn faster. As soon as the network is making better US prediction, uncertainty and ACh concentration decrease, and LA and BAf responses equilibrate. Figure 3E Shows that both LA and BAf weights increase during conditioning.

During extinction, LA firing rates remain stable, BAf activity starts to increase due to ACh effect, then decreases as BAe increases and inhibits BAf. BAe increase of activity also provokes an increase in CeLOff activation, which causes the inhibition of CeLOn activation still powered by LA (Figure 3C). Since no US is presented, only weights to extinction neurons can learn and strengthen (Figure 3F). During renewal, IL is no longer providing extinction signal, which causes BAe to stop firing, and restaures both BAf and CeLOn firing, thus enabling the immediate renewal of the fear response. Figure 3G shows how ACh concentration is changing, depending on uncertainty, and is helping extinction acquisition in BAe. The observed neuronal dynamics of BAf and BAe populations is in full accordance with experiments reported in Herry et al. (2008) and reproduced by the modeling study in Vlachos et al. (2011). Dynamics in LA was not considered in these papers, whereas our experiments suggest a contrasted course of activity in LA, as compared to BA. This will be discussed in Section 4.

**Figure 4:**
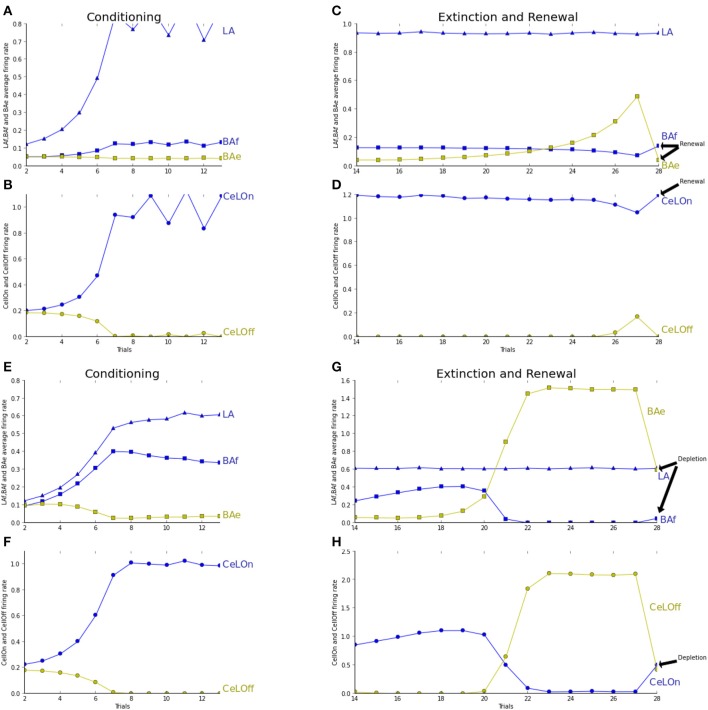
**See caption of Figure 3 for trial description. (A–D)** ACh concentration is fixed to 0.5 for modeling the effect of ACh depletion during all the process of extinction. **(E–H)** Similar to Figure 3. The network is tested in the extinction context with an ACh depletion in the last trial only. The last trial is showing extinction with ACh depletion instead of renewal as in **(A–D). (A)** Fear acquisition is mostly performed by LA increase of activity (blue triangles) during learning, which compensates the weaker learning in BAf (blue squares). BAe activity (green squares) remains steady, since BAf inhibition does not increase. **(B)** CeLOn activity (blue circles) increases, and correctly predicts US arrival at the end of the conditioning period. Thus, conditioning is not impaired by ACh depletion. CeLOff activity (green circles) is inhibited by CeLOn increase of activity. **(C)** LA activities remain high as in non-ACh depleted extinction. BAe activities only increase weakly, which is not sufficient to lower BAf firing rate, which remains steady. **(D)** CeLOn activity does not decrease during extinction, since BAe low increase cannot inhibit LA firing. Extinction learning with ACh depletion is impaired. **(E,F)** Conditioning without ACh depletion, similar to Figure 3. **(G,H)** Extinction without ACh depletion, up to trial 21. In trial 22, ACh is depleted by fixing its level to 0.5, which provokes a decrease in BAe activity. This allows a weak increase in BAf firing rate. BAe decrease also provokes a decrease in CeLOff inhibition, thus enabling CeLOn to fire again. Extinction learning is impaired, yet not suppressed, by ACh depletion.

We tested and report in Figure 4 the effect of ACh depletion in extinction/renewal paradigms. ACh is set to a constant level of 0.5, i.e., 50% of the baseline level. Fear acquisition is not impaired, even if learning essentially takes place in LAf (Figure 4A). Extinction learning is impaired by ACh depletion: BAe neurons takes longer to learn and to overcome BAf inhibition, and so CeLOff cell cannot inhibit CeLOn activity (cf. Figure 4C). To study the effect of ACh depletion not only on learning, but on the competition between LA and BA, as reported in Figures 4G,H, we tested the effect of ACh depletion after extinction training. ACh level is set to 0.5 in the last trial, in the extinction context. BAe activity decreases, which induces a weak increase in BAf, and a strong decrease in CeLOff activity, allowing CeLOn to fire again, thus impairing extinction. Higher levels of ACh depletion (not shown in figures) impair even more extinction memory, which is consistent with results showing that injections of ACh antagonist scopolamine in the amygdala after extinction conditioning impair extinction (Prado-Alcalá et al., 1994).

### 3.2. The role of cholinergic modulation

The study proposed in Calandreau et al. (2006) has also been replicated using our model where, after a classical CS-US pairing, the events are unpaired to mimick a certain level of uncertainty. Depending on levels of ACh (normal or modified), Calandreau et al. (2006) investigates if the CS or the context best predicts the US. In that paper, the unpairing is performed by inserting various delays between CS and US. In our study, we do not experiment variability in time (cf. the concluding section for discussion on implementing PL and other prefrontal structures involved in this kind of processing). Instead, the unpairing experiment is carried out by randomly varying the salience of the sensory CS. Its level of activation is multiplied by a number taken randomly in a uniform distribution between 0 and 1. These limits correspond to the two extreme cases of an absent CS and a paired one.

In the pairing experiment, both CS and CXT are presented in phase 1 and associated to US in phase 2, during 11 trials. Then, the network prediction is tested in a trial with only the sensory CS and a trial with only the context CXT. The procedure is exactly the same in the unpairing experiment, except that the level of activation of the sensory CS is randomly varied along the 11 trials.

**Figure 5:**
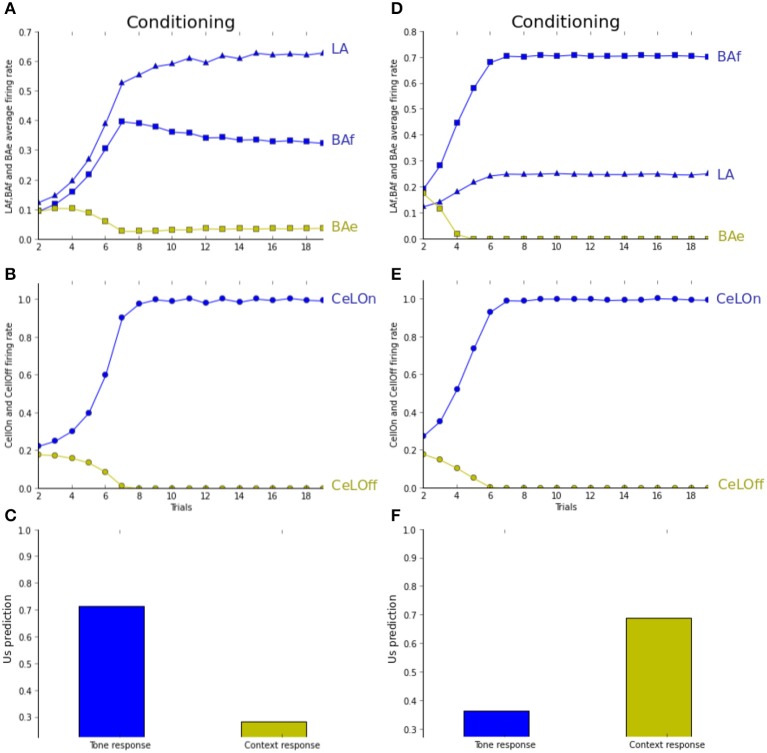
**(A–C)** Pairing experiment (see text for details). **(D–F)** Pairing experiment with increased ACh level. **(B,E)** CeLOn activity increases, and correctly predicts US arrival after a few trials in both experiments. **(A)** LA (blue triangles) and BAf (blue squares) activities increase during training. BAe activities (green squares) weakly decrease due to inhibition. Starting from trial 6, BAf activities are weakly decreasing due to the reduction of uncertainty, which reduces ACh. **(C)** CeLOn activity after learning, when only the tone CS (blue bar) or only the context (green bar) is presented. Prediction is higher for the tone CS, while context-based prediction does not differ much from baseline level (firing rate taken at trial 1, when no CS or context are presented). **(D)** ACh increase causes BAf activities to increase faster than LA activities. **(F)** After learning, CeLOn activity is higher for context-based prediction, and tone-based prediction only shows a weak difference from baseline level. ACh increase causes US to be associated with context instead of CS in the pairing experiment.

Figure 5 reports prediction by the network in the pairing experiment. Pairing under normal conditions is identical to fear conditioning as described previously (Figures 5A,B). Figure 5C shows network prediction, i.e., CeLOn firing rate, following conditioning, if only the tone CS (blue bar) or only the context (green bar) is presented. Context-based prediction does not differ much from baseline firing rate, whereas tone-based prediction is higher. In Figures 5D–F, we report the same experiment with higher level of ACh, by artificially maintaining it to 3.0, which represents around twice the usual level in this experiment. As Figure 5E shows, CeLOn activity is successfully predicting US arrival. It can be observed in Figure 5D that contrarily to normal pairing where learning is distributed between LA and BAf, here BAf is mainly responsible for this prediction. Figure 5F shows that after learning, contrarily to normal pairing, tone-based prediction does not differ much from baseline, whereas context-based prediction is more salient. Our results reproduce (Calandreau et al., 2006) results on the pairing experiment.

**Figure 6:**
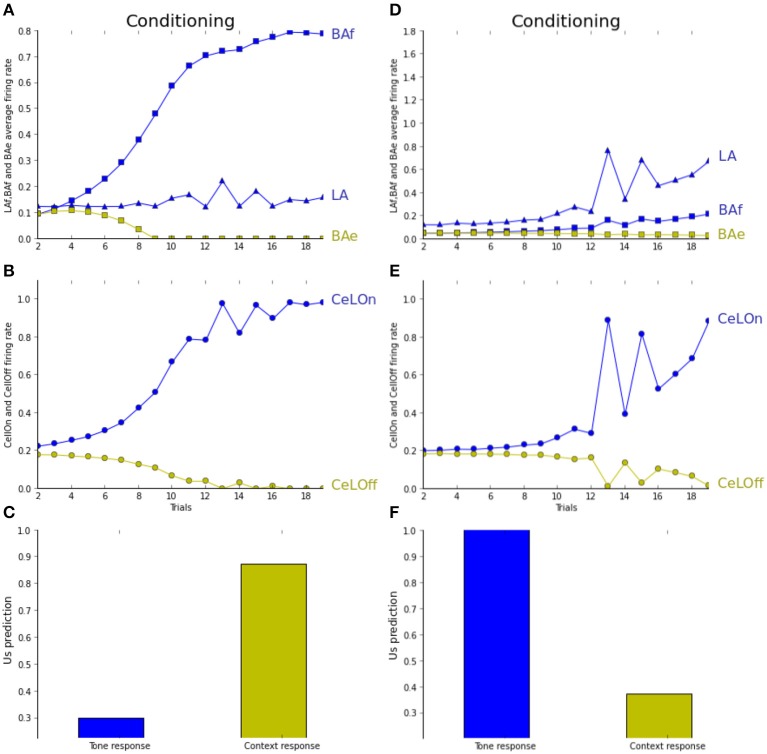
**(A–C)** Unpairing experiment (cf. text for details). **(D–F)** Unpairing experiment with depleted ACh level. **(A)** LA activity (blue triangles) weakly increases when CS is high, whereas BAf activity (blue squares) strongly increases. BAe activity decreases due to BAf inhibition. **(B)** CeLOn activity (blue circles) increases due to BAf increase and predicts US arrival after learning. **(C)** Tone CS alone does not elicit a strong fear response after learning (blue bar), while context does (green bar). **(D)** With ACh depletion, the network is more influenced by CS salience. Activities in both LA and BAf increase during training, comparatively more in LA than in BAf. **(E)** CeLOn activity increases during training, and is correctly predicting US at the end of training when the tone CS is salient. **(F)** After learning, fear response is strong when CS is presented alone, but not when the context is presented alone. ACh depletion causes US to be associated with CS instead of context in the unpairing experiment. Green squares, BAe neurons activity; Green circles, CeLOff neurons activity.

In Figure 6, the network is observed during the unpairing experiment, under normal (A–C) and depleted (D–F) ACh levels. As seen in Figure 6B, the network correctly performs conditioning, mostly based on BAf activities, while LA activities do not increase much during learning (Figure 6A). As a result, tone CS associative strength does not differ from baseline after learning, while context-based prediction is high (Figure 6A). The network mainly learns to associate the context with the US in the unpairing experiment under normal ACh level. Tested with depleted ACh level (ACh maintained to 0.5% of the baseline level during learning), the opposite association is observed. Figure 6E shows that the network is successfully predicting the US when the tone CS is sufficiently salient, and not for lower salience. During learning, activities in LA are increasing quicker than in BAf (Figure 6D). When the tone CS is less salient, both are decreasing. This shows that learning is essentially based on the tone CS. Indeed, Figure 6E shows that context-based CeLOn prediction does not really differ from baseline level, contrarily to tone-based response which is close to one. In short, in both pairing and unpairing experiments, the network is reproducing (Calandreau et al., 2006)'s results on choosing between sensory or contextual cues, depending on CS accuracy and ACh level.

## 4. Discussion

Pavlovian conditioning is a learning paradigm often considered in experimental and modeling works because it is easy to observe and to control and involves well-identified cerebral structures. In the case of somatomotor associations with aversive US and short time interval like eyeblink conditioning, the cerebellum has been identified as the major locus of associative learning (Steinmetz, 2000) and the hippocampus has been shown to provide contextual information (Carretero-Guillén et al., 2013). For stronger US and longer CS-US interval, like in fear conditioning, the amygdala has been shown to perform CS-US association, with a variety of neuronal populations as reported above.

The model proposed in this paper, while simple, allowed us to investigate and identify the precise roles of the main nuclei of the amygdala, with a particular emphasis on their differentiation relatively to MTL and mPFC structures. All the results reported in this paper have been produced by a simple model using point-neurons and rate activation function, with similar equations and parameters for every amygdalar populations. This indicates that properties observed in conditioning protocols emerge from connections and interactions between multiple populations, rather than from complex representations within a population. Experiments carried out with the model were the opportunity to revisit and interpret a series of experiments in the literature, related to fear learning, extinction, fear response and neuromodulation. These elements can also be gathered in a new interpretation of pavlovian conditioning within MTL and its main information flows.

### 4.1. Complementary fear learning in LA and BAf

In the model, neurons in LA and BAf receive external inputs, respectively from the sensory cortex and the hippocampus and learn to anticipate fear. The experiments we have driven have been compared to experiments reported in Herry et al. (2008) and in modeling works by Vlachos et al. (2011). In contrast to this latter study, our model is not only focusing on BA and can be the basis for discussing precisely the respective roles of LA and BAf in fear learning. A strong idea in this regard is to go beyond the impact of external inputs on LA and BAf and to integrate internal relations within the amygdala from LA to BAf. In the pairing experiment, Calandreau et al. (2006) states that the prediction is based on sensory inputs rather than on contextual ones. One could argue that, because the experiment is using a quite salient tone, learning the tone is easier. As an alternative but not contradictory explanation, our model explains this bias toward sensory rules in the pairing experiment with normal ACh level because both LA and BAf receive sensory information (LA directly from the cortex and BAf indirectly from LA) whereas only BAf can predict contextual rules. Tone-based predictions will consequently occur both in LA and BAf and will overcome contextual learning, except for stronger levels of ACh. This interpretation is also consistent with experiments carried out in Herry et al. (2008), involving sensory inputs (simple tones) and reporting activation of BAf neurons.

Results proposed here have been obtained with non-plastic connections from LA to BAf. In experiments non-reported here, identical results were obtained, applying the learning rule on LA-BAf connections. This suggests that LA-BAf learning is not necessary for the different experiments tested here. One hypothesis is that this would be necessary for more precise discrimination, like modulating a specific rule if the strength of the US changes in specific contexts.

One strong feature of our model is that learning is distributed between LA and BAf under normal condition, which is consistent with findings (Anglada-Figueroa and Quirk, 2005) that a lesion of BA after training impairs fear conditioning (because, we argue, a part of the memory trace is in BA), whereas a lesion of BA prior to conditioning does not (because all the memory traces are in LA). Under this view, our model is predicting that a lesion of BA after training under ACh depletion should not, or significatively less, impair fear acquisition.

### 4.2. BAe neurons perform extinction learning

In the model, neurons in BAe learn to extinguish fear responses, based on inputs from IL. Our experiments nicely reproduce the finding that extinction is not forgetting, reporting activities in LA during extinction (Repa et al., 2001; Maren, 2005) and immediate recovery of previous fear response (Herry et al., 2008).

Because our model is mainly a model of the amygdala, we did not implement how an extinction situation is detected in IL. Instead, IL simply transmits a signal indicating an extinction context. In a more realistic approach, this signal should be sent when the recent history contains too many errors to be compatible with the estimated uncertainty of the rule. This challenging problem is the topic of ongoing works.

For the moment, in our model, extinction learning takes place only in BAe. It could be also considered, in accordance with results reported in Anglada-Figueroa and Quirk (2005), that extinction learning could exclusively take place in IL, and no learning would occur in BAe, only relaying IL activity to inactivate fear neurons and activate CeLOff. This seems to us unlikely because as shown in Herry et al. (2008), inactivation of BA completely prevents extinction. After reactivation, animals still exhibit high freezing levels, but are able to learn extinction normally. This implies that no extinction learning occurred in IL while BA was inactive, and seems more in favor of extinction learning taking place in BA, driven by IL indicating or not an extinction context. Moreover, extinction learning is taking more time than acquisition and fear neurons start to decrease their activity after the activity of extinction neurons starts to increase (Herry et al., 2008). Our model reproduces these findings (cf. Figure 3C) and explains these phenomena by fear neurons still predicting US and keeping extinction learning slow in the first trials. Yet this phenomenon would not occur if extinction learning only takes place in IL. Another explanation, non-contradictory with the first one, is that animals need first to learn the extinction context, to differentiate extinction from a stochastic rule, before starting to learn extinction. Yet, both hypotheses are in favor of having an extinction learning in BAe.

Other authors encompass the role of non-amygdalar learning in contextual extinction. Particularly, Anglada-Figueroa and Quirk (2005) report that rats with BA lesions show normal extinction learning. This evidence is nicely reproduced by Moustafa et al. (2013) in his two-process model about the involvement of the hippocampus in fear conditioning. This model incorporates the same regions as in our model, but extinction is implemented with a direct projection from IL to intercalated cells (ITC), which in turn inhibit CeM, with no need for extinction learning in the amygdala. In a more extensive series of experiments, Laurent et al. (2008) reports that rats with BA lesions or BA learning impairment can indeed extinguish a fear response, but do not show extinction if tested the day after, while normal rats do. Both views can be reconciled by considering the involvement of these two pathways at two different time scales (Pauli et al., 2011). The IL-ITC-CeM pathway would be responsible for a rapid flexible learning of extinction whereas the IL-BAe-CeLOff-CeM pathway would perform slow learning and long-term storage. As discussed in Section 4.3, this is a good motivation for reconsidering the role of ITC in our model.

### 4.3. CeLOn neurons prepare the response of the central nucleus

The output of the network is integrated in two different populations of CeLOn and CeLOff neurons, as described in Ciocchi et al. (2010) and reported to integrate the contribution of LA and BA populations in a unique behavioral response. Our model does not assume a similar role for CeLOn and CeLOff. Only CeLOn activity is taken as the output of the model, i.e., we consider that CeLOn activity represents the US prediction of our network. CeLOff will influence this prediction by inhibiting it, but is not a direct output of our model. This is consistent with biological data reported in Ciocchi et al. (2010), showing that whereas both CeLOn and CeLOff project to medial CeA, CeLOn is responsible for the spontaneous burst in medial CeA neurons previous to US arrival, while CeLOff has a more tonic effect. This allows us to explain why it is reported that fear neurons in LA are still firing during extinction (Repa et al., 2001; Maren, 2005): our model proposes that their effect on CeLOn is extinguished by CeLOff inhibition, sustained by BAe.

CeLOff was chosen as the unique locus of inhibition in the model for the sake of simplicity. A more realistic implementation of inhibition of the fear response during extinction would have to consider the respective roles of CeLOff and ITC in inhibiting the fear response in CeM, as we intend to do in future works (Amano et al., 2010; Lee et al., 2013). Another simplification in our model is the absence of plasticity in CeA, even if studies are showing that there is actually plasticity, especially in CeL (Pare et al., 2004). According to our experience, this plasticity should not play a major role in the extinction/renewal or pairing/unpairing experiments. In contrast, this learning may be important to associate direct signals from thalamus to CeA, which is not critical for solving the experiments described here. Such a case is reported in Yu and Dayan (2005), while detecting unknown uncertainty (the rule is not valid, not because of stochasticity but because the rule has changed). In order to find rapidly a new valid rule, the authors explain that it is more efficient to rely on basic cues from the thalamus rather than on high level cues from the cortex, possibly elaborated for the previous rule. The authors suggest that noradrenergic modulation could help biasing the balance between thalamic and cortical inputs to the amygdala, as it is also described in Johnson et al. (2011).

Another possible role for plasticity in CeA could be to learn to weigh multiple afferences to CeL, coming from LA, BA but also IL, to trigger an adapted response, even in complex situations requiring precise discrimination or allowing to generalize behavior (Ciocchi et al., 2010). Here also, this could be the topic of future works, including the design of more complex situations.

### 4.4. Impact of neuromodulation on learning

From a physiological point of view, ACh has been reported to impact neuronal processing by enhancing signal to noise ratio (SNR) in the cortex (Pauli and O'Reilly, 2008), the hippocampus (Hasselmo, 2006) and the basal nucleus of the amygdala (Unal et al., 2015). From a functional point of view, ACh has been proposed to signal known uncertainty (stochasticity) (Yu and Dayan, 2005).

In our model, the impact of ACh is implemented by increasing responses in BA populations proportionally to their levels of activation. On the one hand, this effect actually increases SNR in BA. On the other hand, it favors contextual hippocampal input in BA as compared to learning on sensory cues in LA, which is appropriate in case of known uncertainty. Indeed, in our study adapted from Calandreau et al. (2006), whereas the pairing experiments naturally favor learning on sensory cues in LA, in the unpairing experiments, the stochasticity of the sensory rule provokes an increase in ACh level and results in a quicker learning in BA than in LA. As a result, the contextual rule is learnt instead of the sensory rule.

The main difference between experiments in Calandreau et al. (2006) and in our model is the region where ACh levels are modified (respectively in the hippocampus and in the amygdala). Nevertheless, in both cases, ACh depletion is impairing the same functional pathway concerned with contextual learning and implicitly favors learning on sensory cues. As a prediction, we postulate that performing the same experiments as in Calandreau et al. (2006) with modified ACh levels in BA should deliver similar results, thus comforting this interpretation. This prediction is at some extent supported by McIntyre et al. (2003) reporting a positive correlation between amygadalar ACh and performance in a hippocampus dependent task.

Variation of ACh level in BA has a similar impact on the competition between sensory-based fear prediction and contextual fear extinction. In particular, Schroeder and Packard (2004) show a facilitating effect of intra-amygdalar injection of an ACh agonist in contextual extinction. Prado-Alcalá et al. (1994) report reversal of contextual extinction with ACh antagonist injection. Our model reproduces this reversal with ACh depletion after extinction learning (Figures 4G,H). These findings and those reported just above on contextual vs. sensory learning, highlight the central role of ACh in modulating the different amygdalar pathways. In both cases, by enhancing neuronal activity in BA, increased ACh levels promote alternative solutions to fear response based on sensory cues.

### 4.5. Pavlovian learning within MTL

This integrated view of the amygdala is also a systemic view of pavlovian conditioning in the amygdala in interaction with other cerebral structures. In the aim of learning CS-US associations, at the lowest level of complexity, features characterizing the CS and the US can be simply given by the thalamus and the sensory cortex. In this case, and as it has already been proposed by many models based on the Rescorla-Wagner rule (Rescorla and Wagner, 1972), LA can extract these features by a competitive learning and predict US efficiently. It can also target CeA directly to produce the corresponding pavlovian responses. In the view emerging from our model, BA is involved as soon as the situation goes beyond this standard case and makes the decision from the current evaluation of LA but also from the integration of cues from other cerebral structures.

On the one hand, this is the case when the CS is more complex than in the standard case. The complexity can be in space: the CS can integrate contextual information as we have experimented here or it can correspond to the configural combination of stimuli not present in the cortical representation, as it is for example discussed in O'Reilly and Rudy (2001); in both cases, the hippocampus can learn by heart this configuration and activate BA fear-neurons. In the case of configural representation that our model could also consider in future work, a subsequent consolidation between the hippocampus and the cortex might endow the latter with the needed representation (McClelland et al., 1995); subsequently and as an ultimate goal of pavlovian learning, LA could integrate the association in the scheme of a new simple sensory rule involving cues from the cortex, instead of a specific context from the hippocampus. The complexity can also be in time: a too long CS is extended by a sustained activity in PL, sent to BA as reported in Pendyam et al. (2013); similarly, a delayed CS as considered in Calandreau et al. (2006) might also be represented by a working memory. Extending our model to prefrontal structures would be necessary to extend pavlovian learning to such temporal effect.

On the other hand, the case for more complex association also arises when the CS-US association changes in time, which is not rare in our stochastic and dynamic world. We have evoked here the case of contextual extinction where a rule valid at a certain time becomes suddenly extinguished in another context. We have argued that IL has all the information and functional characteristics to build this history in a working memory and to act on BA accordingly through extinction neurons. The cases where the expected US doesn't arrive because the association is more complex than expected have to be distinguished from the cases where the US doesn't arrive because the rule is simply stochastic and doesn't apply from time to time, just by chance. In this case, there is no concrete new rule to learn, but it must be considered that specific rules involving contextual information should be probably more reliable than general rules based on the presence of sensory cues. This is implemented in our model by the modulatory effect of ACh, as an incitation for preferring contextual rules in BA instead of general rules in LA. ACh release might also result in provoking the learning of such contextual rules in the hippocampus.

In conclusion, this systemic view of the amygdala is also a dynamic view of our memory system, proposing in accordance to many other authors and experiments (McClelland et al., 1995; Holland and Gallagher, 1999; Pauli et al., 2011) that learning in one structure can subsequently improve the memorization process in another structure. An original interpretation of our study proposes such a role for pavlovian conditioning within the MTL, with learning in amygdalar nuclei fed by inputs from the cortex and the hippocampus and enriching subsequently memories in these structures.

### Conflict of interest statement

The authors declare that the research was conducted in the absence of any commercial or financial relationships that could be construed as a potential conflict of interest.
